# Psychological and socio-structural determinants of intentions to use drug checking services

**DOI:** 10.1177/13591053251321783

**Published:** 2025-03-21

**Authors:** Timothy Piatkowski, Kyra Hamilton, Martin S Hagger

**Affiliations:** 1 Griffith University, Australia; 2 Griffith Centre for Mental Health, Australia; 3 Health Sciences Research Institute, University of California, Merced, USA; 4 University of Jyvaskyla, Finland

**Keywords:** behavior change, drug checking, integrated models, social cognition theory

## Abstract

This study explored the determinants of intentions to use drug checking services among Australian undergraduate students (*N* = 324, *M age* = 22.32 years, SD = 7.21) using an integrated theoretical model that includes social cognition constructs (risk perception, subjective norms, attitudes), health and drug literacy, and socio-structural factors (education, race, employment). A cross-sectional correlational survey design and path analysis revealed that social cognition constructs directly influenced drug checking intentions, while drug literacy and socio-structural variables indirectly influenced intentions through these constructs. Notably, race had a negative indirect effect, while drug and health literacy had a positive indirect effect on intentions. The findings highlight the importance of utility beliefs, perceived risks, and social influences in shaping intentions to use drug checking services. These insights offer a foundation for future behavioral interventions targeting belief-based determinants to promote the use of drug checking services, potentially reducing health risks associated with drug use.

## Introduction

Illicit drug use, despite its health and social consequences such as dependence, overdose, and criminalization, persists necessitating efforts to promote safe usage practices. Illicit drug users often lack information on the composition and purity of the drugs they use, in part attributable drug prohibition or regulation, which increases health risks (Miron, 2003; Piatkowski et al., 2024a; Taylor et al., 2016). To address this, drug checking services have been developed as a harm reduction strategy to provide accurate drug information to users (Barratt and Measham, 2022; Maghsoudi et al., 2022). Drug checking services, also referred to as *pill testing* or *adulterant screening*, encompasses comprehensive opportinities that allow recreational drug users to analyze their previously-obtained drugs prior to use and obtain guidance anonymously and without prejudice or legal consequences (Eassey et al., 2024). These services, present for over 50 years and now available in over 20 countries, are either fixed-site services within communities or mobile/on-site services, notably present at events where recreational drug use is more likely (Maghsoudi et al., 2022; Measham, 2019). Exploratory investigations indicate a strong inclination among recreational drug users to utilize drug checking programs (Bardwell and Kerr, 2018; Kennedy et al., 2018; Measham, 2019; Piatkowski and Dunn, 2024). Presently, and despite some national contentious issues around the legal status of drug checking, public safety concerns, and moral debates on drug use (Ritter, 2020), there are two fixed site drug checking services operating in Australia. These specifically exist in the Australian Capital Territory, the capital of Australia, where the CanTEST service has been established (Olsen et al., 2023), and in Queensland where the CheQpoint service is in its trial phase (Australian Broadcasting Coorporation [ABC], 2023) aims to provide similar services by allowing individuals to anonymously submit drug samples for testing and receive harm reduction support.

Despite the potential health and safety benefits of drug users’ use of drug checking services, there is a paucity of research examining users’ propensity to use drug checking services and the factors involved. In particular, identification of the psychological factors linked to service use is important because it may signal potentially-modifiable target variables that can inform the development of persuasive messaging interventions to promote greater service use. Preliminary research in this area has focused on examining users’ attitudes toward drug checking services (Betsos et al., 2022; Caluzzi et al., 2023; Piatkowski et al., 2023, 2024b; Sherman et al., 2019; Wardle et al., 2024). Recent evaluation of the CanTEST service has revealed that the likelihood of using a drug varied significantly based on receiving results from the drug checking service (Olsen et al., 2023). Specifically, if the test results indicate that the drug is not what the individual intended to consume or contains harmful substances, they are more likely to dispose of the drug rather than use it. These studies, however, have not focused on the broader sets of beliefs (e.g. attitudes and norms) that likely relate to behavior and the processes involved, particularly those derived from psychological theory. Alongside this, there is potential to examine the relative effects of psychological factors alongside other service use determinants such as socio-structural variables such as users’ knowledge, education, and socio-economic status and the theory-based processes involved.

### Psychological determinants of drug checking intentions

Research examining the determinants of health-related and safety-oriented behaviors often employs motivational theories from social psychology (Conner and Norman, 2015; Fishbein et al., 2001). These theories operate on the premise that intentions to engage in such behaviors stem from rational decision-making processes informed by social information. Specifically, constructs from these psychological theories influence these decision-making processes and are considered modifiable, thus informing behavior change interventions through targeted techniques (Hagger et al., 2020a; Sheeran et al., 2017). A notable category of these theories is social cognition theories, which posit that individuals’ motivations for engaging in health or safety behaviors depend on their beliefs about performing those behaviors in the future (Conner and Norman, 2015; Hagger and Hamilton, 2022). Social cognition theories have effectively predicted intentions for various health-related behaviors, including hand sanitizing, sun safety, and dental care (Conner and Norman, 2015; Hagger and Hamilton, 2022). Prominent theories, such as the Theory of Planned Behavior (Ajzen, 1991) and Protection Motivation Theory (Rogers, 1975), identify intention and other related constructs (e.g. protection motivation) as key determinants of health behavior (Fishbein et al., 2001). Intention is shaped by a constellation of belief-based constructs: attitudes (evaluations of the usefulness of engaging in a behavior), subjective norms (perceived influences of significant others), perceived behavioral control or self-efficacy (confidence in the ability to perform the behavior), and risk perceptions (perceived personal risk associated with the behavior). Meta-analyses have consistently supported the relationships between these constructs and intentions to engage in health behaviors across various populations and contexts (McEachan et al., 2011; Milne et al., 2000). These constructs have also demonstrated efficacy in testing relationships between drug use and engagement with treatment services.

Research utilizing the social cognition models has demonstrated its relevance in predicting treatment engagement post-detoxification. A pilot study with 168 participants from Salvation Army detoxification units in Australia found that attitudes and perceived behavioral control significantly influenced intentions to pursue further treatment (Kelly et al., 2011). Additionally, a study of 400 African-American cocaine users identified treatment effectiveness and readiness for treatment as key predictors of perceived need for intervention (Booth et al., 2014). An extended TPB framework in a Norwegian trial showed that attitudes and perceived control explained 81% of the variance in intentions to attend 12-step groups, highlighting the importance of psychological constructs in enhancing participation in drug services (Vederhus et al., 2015). These findings underscore the potential for targeted interventions to improve engagement with drug services.

In the context of drug checking services, these theories suggest that intentions to utilize such services may similarly be influenced by users’ attitudes toward drug safety, perceived norms surrounding drug use, and their confidence in the ability to make informed choices. By critically applying these frameworks to drug checking, we can identify specific belief-based constructs that shape intentions to use drug checking services, thereby informing targeted interventions. This examination of social cognition models highlights the importance of understanding how intentions to engage in drug checking are formed and what factors may be leveraged to promote service uptake among users. In one example, Davis and Rosenberg (2016) applied the theory of planned behavior to predict drug checking service use intentions and behavior among ecstasy users. Consistent with the theory, findings indicated that attitudes, subjective norms, and perceived behavioral control were associated with the intention to use these services, with the small-to-medium sized effects, and intention the sole predictor of subsequent follow-up use of the checking service 2 months later, with a small effect size. This research provides preliminary evidence of the psychological determinants of drug checking service use and paves the way for future applications of social cognition theories to identify the correlates of drug checking intentions and behavior more broadly.

Although social cognition theories are useful for identifying psychological determinants in decisions to use drug checking services, these should be considered alongside socio-structural factors like demographic characteristics (e.g. age, gender) and socio-economic background (e.g. income, education), which have been linked to health behavior disparities. For example, lower income and education levels are associated with reduced participation in health behaviors (Stringhini et al., 2010). Examining social cognition alongside socio-structural variables can reveal not only the unique determinants of intentions but also how these factors influence behavior. Studies show that socio-structural variables indirectly affect intentions through belief-based constructs (Godin et al., 2010; Hagger and Hamilton, 2022), such as education influencing intentions via attitudes and perceived behavioral control. This suggests that individuals with lower education may lack the knowledge or skills needed to perform health behaviors, reducing their intentions to do so.

Alongside socio-structural determinants of health behaviors representing socio-economic status and social environment (e.g. income, education), researchers have identified individuals’ health literacy as a further highly relevant determinant of intentions to perform health behaviors such as physical activity, smoking, and alcohol consumption (McAnally and Hagger, 2023). Health literacy reflects individuals’ capacity to comprehend and utilize health information to make decisions that are relevant to health, and has been shown to be an important correlate of health-related decision making and health outcomes across multiple contexts such as hand washing and vaccination (Kim et al., 2022; Rolova et al., 2021). The comparator variable in the context of drug use and drug checking behavior is drug literacy, which reflects individuals’ comprehension of specific knowledge about recreational drugs and their effects, particularly side and health effects, and their capacity to apply that knowledge to their own decisions on drug use. We posit drug literacy is crucial as it directly influences individuals’ intentions to engage with drug checking services; higher drug literacy may lead to more informed choices and safer behaviors. Health literacy, and specific forms of health-related literacy such as drug literacy, has been shown to be consistently linked with health-related behavior (Kim et al., 2022).

Importantly, consistent with research examining the processes by which other socio-structural variables relate to health behavior, effects of health literacy on intentions and health behavior are expected to be mediated by the social cognition constructs (Hagger and Hamilton, 2022). Health literacy represents individuals’ health behavior knowledge and capacity to use it and is therefore likely to serve an informational function when individuals estimate the utility of, and their capacity to perform, a given health behavior. Prior research has corroborated these mediation effects. Specifically, a meta-analysis of research on relations between health literacy, social cognition constructs, and health behaviors not only supported associations among these constructs across multiple studies but also provided evidence supporting the proposed mediation effect (McAnally and Hagger, 2023). By extension, this pattern of effects may operate when it comes to the effects of drug literacy on intentions to use drug checking service among drug users.

### The present study

Accordingly, the aim of this study was to extend preliminary evidence relating to drug users’ perspectives on drug checking services (Barratt et al., 2018; Wardle et al., 2024) by identifying the salient social cognition and socio-structural determinants of users’ intentions to use drug checking services and the psychological mechanisms involved. Specifically, we aimed to test effects of users’ attitude, subjective norms, perceived behavioral control, and risk perception with respect their future use of checking services on their intentions to use these services consistent with social cognition theories (Ajzen, 1991; Fishbein et al., 2001; Rogers, 1975). Concurrently, we also aimed to examine effects of a panel of socio-structural variables representing users’ socio-economic status (race, employment, education) and the extent of their knowledge of drugs and its application (health and drug literacy) on intentions, and the mechanisms involved. Specifically, we aimed to examine the indirect effects of these variables on intentions mediated by social cognition constructs consistent with prior theory and research

**H1:** Attitudes, subjective norms, perceived behavioral control, and risk perceptions will have significant direct effects on intentions to use drug checking services.
**H2:** Socio-structural variables, including race, employment status, education level, and drug literacy, will have significant indirect effects on intentions to use drug checking services, mediated by the social cognition constructs.

In terms of specific predictions, we aimed to test the fit of a structural model specifying simultaneous associations among the social cognition constructs, socio-structural variables, and intentions to use drug checking services in data collected from a sample of Australian recreational drug users. In keeping with prior research applying social cognition theories in health behavior contexts (Conner and Norman, 2015; Hagger and Hamilton, 2022; Hamilton et al., 2020; McAnally and Hagger, 2023), we expected unique effects of users’ attitudes, subjective norms, perceived behavioral control, and risk perceptions on their service use intentions. This suggests that the way users think about drug checking, the social pressures they feel, their confidence in accessing these services, and their understanding of potential risks will each uniquely shape their willingness to engage with drug checking services. Further, we predicted that effects of a set of socio-structural variables representing socio-demographic (race), socio-economic status (employment, education), and knowledge use (health and drug literacy) on drug checking use intentions would be mediated by the social cognition constructs (attitudes, norms, risk perceptions, and perceived behavioral control).

## Method

### Participants and design

The present study employed a cross-sectional correlational survey design. Participants were University students (*N* = 324, 65.85% female) recruited from the University online research system portal. To be eligible for inclusion, participants had to be aged 18 years or older. Data were collected between July and October 2023.

### Procedure

Eligible participants were informed, prior to initiating the survey, that they were being asked to participate in a survey on drug checking attitudes and intentions. Subsequently, they were provided with information outlining study requirements, informed of their right to decline participation at any point, and required to provide opt-in informed consent to participate prior to advancing to the survey. Consenting participants completed self-report measures of social cognition constructs from the proposed extended social cognition model (attitude, subjective norm, perceived behavioral control, risk perceptions) and intentions with respect to utilizing a drug checking service in future, and measures of health literacy and drug literacy. Participants also self-reported a series of socio-demographic variables. Data were collected using the Qualtrics™ online survey platform. Approval (2023/512) for study procedures was granted prior to data collection from the Griffith University Research Ethics Committee.

### Survey measures

#### Social cognition constructs

Multi-item measures of the attitude, subjective norm, perceived behavioral control, risk perceptions, and intention constructs from the proposed social cognition model were developed according to published guidelines (Ajzen, 1991; Schwarzer et al., 2008) with responses provided on 7-point response scales. Each measure referenced the target behavior of utilizing a drug checking service in future. Complete study measures are provided in Appendix A (Supplemental Materials).

#### Socio-structural variables

Participants self-reported a number of socio-demographic variables: age in years; sex (male, female, non-binary); employment status (currently unemployed/full time caregiver, part-time/casual employed, currently employed full-time, leave without pay/furloughed, retired); race (Black, Caucasian/White, Asian, Middle-Eastern, other); highest level of formal education (completed junior/lower/primary school, completed senior/high/secondary school, post-school vocational qualification/diploma, undergraduate university degree, postgraduate university degree); and lifetime drug use (alcohol, tobacco, cannabis, amphetamine, cocaine, opioids, inhalants, sedatives, hallucinogens, anabolic steroids). Dichotomous highest education level (completed school education only vs completed post-school education), employment status (full-time/part-time/casual vs leave without pay, currently unemployed), and race (Caucasian/White vs non-White) variables were computed for use in subsequent data analyses.

#### Health literacy

This measure comprised two-items which represented comprehension (“When you have a medical or health issue, how often do you have problems understanding information you find, or is provided to you by your doctor, about that issue?”) and confidence (“How confident are you filling out health information forms or medical information forms by yourself?”) with respect to medical- and health-related matters, respectively. Responses to these items were provided on 7-point scales with “never”-“always” and “not at all confident”-“extremely confident” scale endpoints, respectively.

#### Drug literacy

We developed a drug literacy scale for the present study based on prior developmental research on health literacy measurement. Drug literacy was defined as a context-specific form of health literacy and based on prior research on health literacy. Participants were asked to assess their confidence in utilizing available health-related information about recreational drugs they consume to make decisions on their future drug use (e.g. whether or not to use, dosage). The questions were subdivided into four domains: overall safety (e.g. overdose potential); health effects (e.g. headaches, vomiting); effects on the body (e.g. balance, coordination); and effects on social and cognitive skills (e.g. communication, speech, perception, judgment) with responses provided on 5-point scales (“not at all” and “extremely”).

### Data analysis

Proposed effects in our extended model were tested using path analysis with robust standard errors (1000 bootstrapped resamples) and a maximum likelihood estimation method. To examine Hypothesis 1 (H1), the model specified direct effects the social cognition constructs (attitude, subjective norm, perceived behavioral control, risk perceptions) on intention to use drug checking services, and direct effects of socio-structural variables (race, employment status, education level, health literacy, drug literacy) on each social cognition construct. To test Hypothesis 2 (H2), we also estimated specific indirect effects of each socio-structural variable on intention mediated by each social cognition construct. Each set of socio-structural and social cognition predictor variables were set to correlate. We also included age and sex as additional covariates in the analysis by including direct effects of each variable on each socio-structural variable and social cognition construct. Missing data were treated using a full-information maximum likelihood (FIML) imputation method.

We evaluated overall fit of the proposed model with the data using multiple recommended indicates of fit. As the goodness-of-fit chi-square (χ^2^) comparing researcher-imposed models with the fully-free model is typically oversensitive to mis-fit, we used a series of incremental fit indices that have been suggested as more viable alternatives to estimate model fit: the comparative fit index (CFI), the standardized root mean-squared of the residuals (SRMR), and the root mean square error of approximation (RMSEA) and its 90% confidence interval (90% CI). Based on simulation studies, values for the CFI that approach or exceed 0.95, values for the SRMR that are less than or equal to 0.08, and values for the RMSEA approaching or below 0.06 with a narrow 90% CI indicate acceptable model fit (Hu and Bentler, 1999). The model also enabled tests of specific direct effects and indirect effects with accompanying sums of indirect effects proposed in our hypothesized model. Specifically, we tested the direct effects predicted in H1, while the indirect effects predicted in H2 were examined using standardized parameter estimates (β) for each with accompanying 95% confidence intervals (95% CI) and one-tailed *z*-tests to provide a formal estimate of difference from the null. Analyses were implemented using the lavaan package in R (Rosseel, 2012) using a maximum likelihood estimator with bootstrapped standard errors derived from 1000 sample redraws.

## Results

### Participant characteristics

Of the 360 responses received, 324 participants (*M* age = 2.32 years, SD = 7.21; male *n* = 104, female *n* = 214, non-binary *n* = 6) provided complete data sufficient for analysis. Thirty-six participants either initiated the survey or did not complete all parts. A majority of the sample was highly educated (*n* = 203, 62.7% completed senior/high/secondary school), typically engaged in casual or part-time work (*n* = 242, 74.7%), and identified as Caucasian/White (*n* = 239, 73.8%). Substantive proportions of the sample reported using tobacco products (*n* = 85, 25.9%; cigarettes, chewing tobacco, cigars), alcoholic beverages (*n* = 144, 43.9%; beer, wine, spirits), and recreational drugs such as cannabis (*n* = 89, 27.1%), cocaine and derivatives (*n* = 37, 11.3%; coke, crack), amphetamine types of stimulant (*n* = 38, 11.6%; speed, diet pills, ecstasy), and hallucinogens (*n* = 40, 12.2% LSD, acid, mushrooms, PCP, ketamine). Full details of sample demographic and drug use characteristics are reported in the table in Appendix B (see Supplemental Materials).

### Preliminary analyses

Descriptive statistics, internal consistency coefficients or inter-item correlations, and inter-correlations for composite constructs are presented in Table 1. The variables investigated demonstrated satisfactory internal consistency (Cronbach α range = 0.774–0.958) or inter-item correlations. The only exception was the health literacy scale, with a low inter-item correlation (*r* = 0.113), which is similar to levels observed elsewhere (McAnally and Hagger, 2023) – results using this variable should be interpreted with due consideration of these inadequate reliability statistics. The drug literacy scale exhibited adequate internal consistency (α = 0.96) and psychometric properties (please see Appendix C, Supplemental materials for details).

**Table 1.** Descriptive statistics and zero-order correlations among study variables.

Variable	*M*	SD	1	2	3	4	5	6	7	8	9	10	11	α
1. Age	22.367	7.245	–											–
2. Sex	–	–	−0.108	–										–
3. Race	–	–	−0.023	−0.023	–									–
4. Employment	–	–	−0.082	−0.088	−0.231**	–								–
5. Education	–	–	0.354***	−0.052	0.018	0.009	–							–
6. Intention	4.911	1.788	0.122*	−0.041	−0.164**	0.077	0.075	–						0.932
7. Attitude	5.454	1.304	0.011	0.024	−0.267***	0.014	0.100	0.506***	–					0.888
8. SN	5.208	1.431	0.018	0.047	−0.232***	−0.017	0.031	0.550***	0.566***	–				0.877
9. PBC	5.912	1.037	0.010	0.026	−0.216***	0.054	−0.025	0.329***	0.403***	0.424***	–			0.774
10. Risk perception	2.503	1.089	0.040	−0.023	0.092	0.092	0.017	−0.023	−0.200***	−0.192**	−0.156**	–		.769^a^
11. Drug literacy	3.973	0.958	0.026	0.023	−0.123*	0.033	−0.073	0.119*	0.256***	0.135*	0.250***	−0.089	–	0.958
12. Health literacy	3.951	0.833	0.310***	−0.103	−0.201***	−0.051	0.152**	0.239***	0.190**	0.151**	0.238***	−0.022	0.250***	.113^a^

*N* = 324.

α: Cronbach alpha reliability coefficient; SN: subjective norm; PBC: perceived behavioral control.

a Reliability coefficient is inter-item correlation as computing an alpha coefficient is contra-indicated with two-item scales.

* *p* < 0.05. **p < 0.01. ***p < 0.001.

### Path analytic model

**Figure 1.**
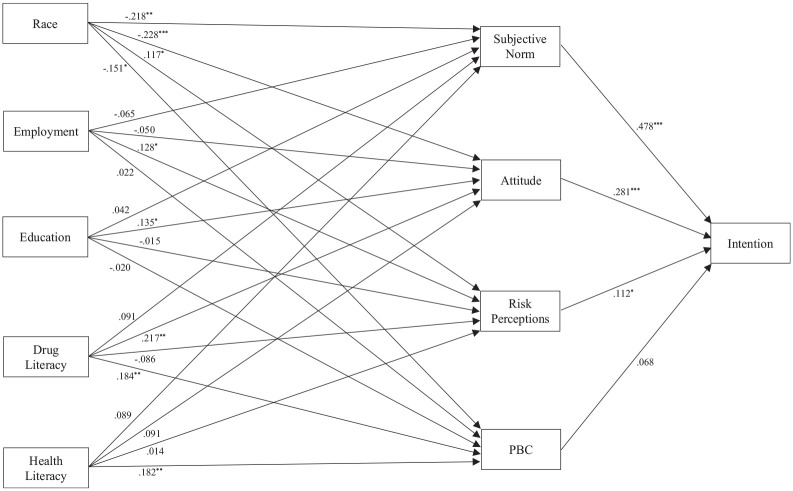
Results of path analytic model predicting drug checking service use intentions. *Note*. Exogenous variables and covariates, and error terms of endogenous predictor variables, were freely correlated. Effects of age and sex covariates on all study variables not shown. PBC = perceived behavioral control; Education = dichotomized education level variable; Employment = dichotomized employment status variable; Race = dichotomized race variable. ***p < 0.001 **p < 0.01. *p< 0.05.

The path analytic model exhibited acceptable fit with the data according to the multiple criteria adopted, χ^2^ (8) = 21.029, *p* = 0.007, CFI = 0.977, RMSEA = 0.071, 90% CI RMSEA [0.035, 0.109], SRMR = 0.031. Standardized parameter estimates and 95% confidence intervals for proposed model effects are presented in Table 2 and summarized in Figure 1. Consistent with hypotheses (H1), we observed non-zero direct effects of attitude, subjective norms, and risk perceptions on drug checking service use intentions. However, the direct effect of perceived behavioral control on intention was no different from zero. In addition, we observed non-zero positive direct effects of health and drug literacy on perceived behavioral control, and of drug literacy on attitude. Further, there were non-zero negative effects of race on attitudes, subjective norms, and perceived behavioral control, and a positive effect on risk perceptions. Further, we found non-zero positive effects of employment on risk perceptions, and of education on attitude. Consistent with hypotheses (H2) we also observed non-zero negative indirect effects of race on drug checking service intentions mediated by attitude and subjective norms, and positive indirect effects of education and drug literacy on intentions mediated by attitude. These indirect effects translated into a non-zero negative sum of indirect effects of race, and a positive sum of indirect effects of drug literacy, on intentions. Interestingly, although all specific indirect effects of health literacy on intention were no different from zero, we observed a non-zero positive sum of indirect effects of health literacy on intention—once the small specific indirect effects of health literacy on intention through all social cognition constructs were totaled the sum was sufficiently large to exhibit a confidence interval that did not encompass zero. Overall, the model accounted for substantive variance in drug checking intentions (*R*^2^ = 0.403).

**Table 2.** Standardized parameter estimates and variability estimates for direct and indirect effects of estimated path analytic model.

Effects	β	95% CI	
		LL	UL
Direct effects			
Attitude→Intention	0.281^***^	0.162	0.399
SN→Intention	0.378^***^	0.258	0.497
PBC→Intention	0.068	−0.026	0.163
RP→Intention	0.112^*^	0.023	0.201
Race→Attitude	−0.238^***^	−0.358	−0.118
Race →SN	−0.218^***^	−0.338	−0.098
Race →PBC	−0.151^**^	−0.266	−0.037
Race →RP	0.117^*^	0.000	0.234
Employment→Attitude	−0.050	−0.134	0.035
Employment→SN	−0.065	−0.169	0.038
Employment→PBC	0.022	−0.089	0.133
Employment→RP	0.128^*^	0.016	0.239
Education→Attitude	0.135^*^	0.024	0.246
Education→SN	0.042	−0.072	0.156
Education→PBC	−0.020^*^	−0.139	0.099
Education→RP	−0.015^*^	−0.132	0.102
DL→Attitude	0.217^**^	0.091	0.342
DL→SN	0.091	−0.050	0.233
DL→PBC	0.184^***^	0.056	0.312
DL→RP	−0.086	−0.210	0.038
HL→Attitude	0.091	−0.034	0.215
HL→SN	0.089	−0.031	0.208
HL→PBC	0.182^***^	0.061	0.302
HL→RP	0.014	−0.109	0.137
Indirect effects			
Race→Attitude→Intention	−0.067^**^	−0.111	−0.023
Employment→Attitude→Intention	−0.014	−0.039	0.011
Education→Attitude→Intention	0.038^*^	0.005	0.071
DL→Attitude→Intention	0.061^**^	0.019	0.102
HL→Attitude→Intention	0.025	−0.012	0.063
Race→SN→Intention	−0.082^**^	−0.028	0.060
Employment→SN→Intention	−0.025	−0.064	0.015
Education→SN→Intention	0.016^*^	−0.028	0.060
DL→SN→Intention	0.035	−0.019	0.089
HL→SN→Intention	0.033	−0.013	0.080
Race→RP→Intention	0.013	−0.004	0.031
Employment→RP→Intention	0.014	−0.003	0.032
Education→RP→Intention	−0.002	−0.015	0.012
DL→RP→Intention	−0.010	−0.026	0.007
HL→RP→Intention	0.002	−0.012	0.016
Race→PBC→Intention	−0.010	−0.025	0.005
Employment→PBC→Intention	0.002	−0.006	0.009
Education→PBC→Intention	−0.001	−0.010	0.007
DL→PBC→Intention	0.013	−0.006	0.031
HL→PBC→Intention	0.012	−0.008	0.033
Sums of indirect effects^a^			
Race→Intention	−0.146^***^	−0.225	−0.068
Employment→Intention	−0.023	−0.081	0.036
Education→Intention	0.051	−0.016	0.118
DL→Intention	0.098^*^	0.010	0.186
HL→Intention	0.073^*^	0.000	0.146
Effects of covariates			
Age→Intention	0.099^*^	0.016	0.183
Age→Attitude	−0.078	−0.182	0.026
Age→SN	−0.032	−0.162	0.097
Age→PBC	−0.042	−0.173	0.088
Age→RP	0.056	−0.073	0.184
Sex→Intention	−0.053	−0.139	0.032
Sex→Attitude	0.016	−0.093	0.125
Sex→SN	0.041	−0.066	0.148
Sex→PBC	0.032	−0.074	0.138
Sex→RP	0.003	−0.112	0.118

β: standardized parameter estimate; 95% CI: 95% confidence interval of standardized parameter estimate; LB: lower bound of 95% CI; UB: upper bound of 95% CI; SN: subjective norms; PBC: perceived behavioral control; RP: risk perceptions; DL: drug literacy; HL: health literacy; Race: dichotomized race/ethnicity variable; Employment: dichotomized employment status variable; Education: dichotomized education level variable.

a Sum of indirect effects of through all model constructs. Exogenous variables and covariates, and error terms of endogenous predictor variables, were freely correlated.

*** *p* < 0.001. **p < 0.01. *p < 0.05.

## Discussion

The present study aimed to identify the psychological and socio-structural determinants of undergraduate students’ intentions to use drug checking services, focusing on constructs from social cognition theories (attitudes, subjective norms, perceived behavioral control, risk perceptions) and variables like race, education, employment, health literacy, and drug literacy. Testing an integrated model, results revealed that attitudes, subjective norms, and risk perceptions directly influenced intentions, while race, education, and drug literacy had indirect effects through these social cognition constructs. Additionally, race, drug literacy, and health literacy also had indirect effects on intentions mediated by social cognition constructs.

This study offers preliminary insights into the psychological constructs and socio-structural variables influencing students’ intentions to use a drug checking service, consistent with established theories, and highlights a potential underlying mechanism. Focusing first on the direct, theory-based determinants of intentions, these findings suggest that attitudes and subjective norms had the largest effects on intentions, with a smaller but notable role for risk perceptions. This indicates that students’ beliefs regarding the usefulness and positive outcomes of drug checking, along with their perceptions of important social influences, strongly shape their intentions to use such services. These findings are consistent with prior research on health behaviors like vaccine uptake (Ampt et al., 2009; Ng et al., 2020) and engagement with drug and alcohol services (Kelly et al., 2011), where beliefs in the utility of the behavior and perceived social influences are key predictors of intentions. The smaller effect of risk perceptions suggests that while students are aware of the risks associated with drug use (Blood et al., 2003), these risks may seem less salient potentially due to a range of factors, such as limited direct experience or varying perceptions of drug danger based on classification (e.g. marijuana vs heroin), making them more abstract compared to attitudes and norms. Moreover, despite the potential influence of drug checking service availability at the time of data collection, perceived behavioral control was not a significant determinant of intentions. This may be due to the hypothetical nature of the behavior as presented in the survey, as well as potential geographical barriers. For example, participants may have perceived limited access to drug checking services if those facilities were located far from their residential location, making the behavior seem less feasible in practice.

An important and unique contribution of the current study was the examination of the effects of the social cognition constructs on drug checking intentions alongside effects of socio-structural factors and, importantly, testing a proposed theory-based mechanism for these effects. The direct effects of these factors provided some basis for individuals reporting of their drug checking intentions. Specifically, we observed direct effects of key factors on individuals’ intentions. For example, race was associated with more negative attitudes, subjective norms, and perceived behavioral control, which may suggest that individuals from certain racial backgrounds experience greater social or structural barriers that shape more negative views or lower perceived ease of access to drug checking services. In contrast, education was linked to more positive attitudes toward drug checking, while both drug and health literacy positively influenced attitudes and perceived control over using these services. However, for drug literacy, the effect on attitudes was less certain because the confidence intervals included zero, meaning that this effect was small and could potentially be due to chance. Taken together, these findings confirm the prediction from social cognition theories that these factors serve an informational function. Individuals tend to draw on these constructs when estimating their health behavior intentions toward drug checking (Ajzen, 1991; Kaushal et al., 2021). These findings highlight important patterns in students’ decision-making. Non-white students were more likely to report lower beliefs in the utility of drug checking services, less support from others, and less confidence in their ability to use the services, while perceiving higher risks compared to white students. This aligns with research showing that individuals from underserved minority groups often report fewer positive beliefs about engaging in health behaviors, possibly due to experiences of prejudice and disenfranchisement from healthcare services (McAnally and Hagger, 2023). Similarly, students with lower education levels and less drug literacy were less likely to see the utility and benefits of drug checking and expressed lower confidence in using such services. This may reflect a lack of sufficient health education or behavioral skills, preventing them from understanding the links between health and behavior or how to engage effectively with health services.

From a practical perspective, these findings highlight key constructs that could serve as targets for public health interventions aimed at promoting the uptake of drug checking services. Consistent with theory and prior research in health behavior (Conner and Norman, 2015; Lin et al., 2020), beliefs about utility, norms, and risk play a critical role in shaping students’ intentions, partially explaining the associations between socio-structural variables and health behavior disparities. These findings contribute to the literature by demonstrating that minority groups and individuals with lower drug literacy are less likely to perceive the utility of drug checking services or feel supported in using them—insights that extend prior work by identifying specific belief patterns related to drug checking behavior that have been underexplored in the empirical literature (Davis and Rosenberg, 2016). This suggests that interventions targeting these modifiable beliefs, as supported by behavior change research (Hagger et al., 2019, 2020b), could be effective in influencing intentions to use drug checking services. Furthermore, the present study adds nuance to existing models of health behavior by showing that the perceived risks of drug checking vary based on socio-structural factors like race and education, highlighting how these perceptions can amplify or diminish intentions to use harm reduction services. While structural changes addressing economic and educational disparities are essential for long-term solutions, tailored interventions focusing on beliefs related to drug checking service use—especially among minority groups and those with lower health and drug literacy—could have a more immediate impact. For example, promoting the utility of drug testing and addressing misconceptions could enhance intentions among at-risk users. This underscores the need for public health campaigns to be more culturally sensitive and inclusive, adapting messaging to address the unique concerns of underserved groups. Evidence from CanTEST supports this approach, showing that users are more likely to discard drugs when test results reveal unexpected or dangerous substances (Olsen et al., 2023). However, we caution against drawing definitive conclusions or recommending specific interventions based on these preliminary findings, advocating for further research to validate and extend our results.

### Contribution, limitations, and avenues for future research

The current study is the first to examine relations between social cognition constructs and socio-structural variables and intentions to use drug checking services and the theory-based mechanism involved. Findings provide preliminary evidence for theory-consistent patterns of association among social cognition constructs, socio-structural variables, and drug checking use intentions in a sample of undergraduate students, particularly identifying the importance of attitudes, subjective norms, and risk perceptions as having unique effects on drug checking use intentions and indirect effects of education, race, and health and drug literacy on drug checking use intentions mediated by social cognition constructs. The present findings provide a signal as to the potentially modifiable constructs that may be targeted in behavioral interventions using techniques such as persuasive communication purposed to promote intentions to use drug checking services.

However, it is important to note some limitations against which the current findings should be interpreted. First, the current sample of university undergraduate students was not recruited using random selection or stratified by socio-structural variables. As such the current findings cannot be generalized to the broader undergraduate student population or further to the general population. So, effectively, the current study provides an initial set of findings testing these hypotheses that should signal potential effects and mechanisms that are in need of further corroboration. A limitation of this study is that we only assessed lifetime recreational drug use, which does not allow us to distinguish between frequent users and those who may have only used a drug once. Future studies should seek to replicate the current model in samples more representative of the general population. Importantly, given the current sample was also relatively homogenous in racial and ethnic background, age, income, employment, and educational level, there is a need for studies on more diverse populations that permit better extrapolation of the current effects in underserved subgroups such as groups of individuals with lower education levels, on lower incomes, and from minority racial and ethnic backgrounds. Second, the cross-sectional, correlational design of this study limits any inference of causality among social cognition constructs, socio-structural variables, and drug checking intentions. The direction of effects is inferred from theory, not data, meaning other models may fit statistically but lack conceptual validity. Additionally, unmeasured variables could influence the estimated effects. Future research should use cross-lagged panel designs or experimental studies to test these effects more robustly. Despite these limitations, our findings offer insights into the size of effects and unique variance shared with drug checking intentions, providing a foundation for future research. Finally, the absence of a direct measure of drug checking in Queensland highlights the need for future studies to examine actual behavior, as intentions do not fully predict behavior. Research into the intention-behavior link could inform strategies to promote behavior enactment, such as action and coping planning (Hagger and Luszczynska, 2014).

## Conclusions

Drug checking initiatives are expected to play an important role in harm reduction for illicit drug consumers. The effectiveness of these services relies on adoption of these services and behavioral interventions may be one means that public health departments and harm prevention services opt to employ in order to promote service use among users. Accordingly, the present study provided some initial evidence to identify potential determinants of intentions to use drug checking service, including belief-based psychological constructs from social cognition theories that are proposed to underpin decisions to use services and salient socio-structural variables that may affect users’ beliefs and intentions. The findings signal the belief-based constructs, particularly attitudes and subjective norms, alongside perceptions of risk, that are uniquely associated with intentions to use drug checking services. They also indicate that these beliefs are also implicated in the association between socio-structural variables, particularly race, education level, and health and drug literacy, and intentions. These provide an initial signal of the determinants of intentions to use these services in a sample of undergraduate students. However, we underscore the preliminary nature of this research and advocate for future research that not only validates current findings using study designs that enable causal inference, but also extends them to predict actual usage and more representative populations.

## Supplemental Material

sj-docx-1-hpq-10.1177_13591053251321783 – Supplemental material for Psychological and socio-structural determinants of intentions to use drug checking services Supplemental material, sj-docx-1-hpq-10.1177_13591053251321783 for Psychological and socio-structural determinants of intentions to use drug checking services by Timothy Piatkowski, Kyra Hamilton and Martin S Hagger in Journal of Health Psychology

## References

[bibr1-13591053251321783] Ajzen I (1991) The theory of planned behavior. Organizational Behavior and Human Decision Processes 50(2): 179–211.

[bibr2-13591053251321783] Ampt AJ Amoroso C Harris MF , et al. (2009) Attitudes, norms and controls influencing lifestyle risk factor management in general practice. BMC Family Practice 10(1): 8. 19706198 10.1186/1471-2296-10-59 PMC2746183

[bibr3-13591053251321783] Australian Broadcasting Coorporation [ABC] (2023) Queensland government announces pill testing trial at fixed and mobile sites. Available at: https://www.abc.net.au/news/2023-02-25/queensland-pill-testing-drug-law-reform/102022942

[bibr4-13591053251321783] Bardwell G Kerr T (2018) Drug checking: A potential solution to the opioid overdose epidemic? Substance Abuse Treatment Prevention and Policy 13(1): 1–3. 29801458 10.1186/s13011-018-0156-3 PMC5970470

[bibr5-13591053251321783] Barratt MJ Bruno R Ezard N , et al. (2018) Pill testing or drug checking in Australia: Acceptability of service design features. Drug and Alcohol Review 37(2): 226–236. 28635057 10.1111/dar.12576

[bibr6-13591053251321783] Barratt MJ Measham F (2022) What is drug checking, anyway? Drugs, Habits and Social Policy 23(3): 176–187.

[bibr7-13591053251321783] Betsos A Valleriani J Boyd J , et al. (2022) Beyond co-production: The construction of drug checking knowledge in a Canadian supervised injection facility. Social Science & Medicine 314: 115229. 36274456 10.1016/j.socscimed.2022.115229

[bibr8-13591053251321783] Blood RW Williams J McCallum K (2003) Representations of public risk: Illegal drugs in the Australian press. Media Information Australia 108(1): 82–100.

[bibr9-13591053251321783] Booth BM Stewart KE Curran GM , et al. (2014) Beliefs and attitudes regarding drug treatment: Application of the theory of planned behavior in African-American cocaine users. Addictive Behaviors 39(10): 1441–1446. 24930051 10.1016/j.addbeh.2014.05.012 PMC4123798

[bibr10-13591053251321783] Caluzzi G Torney A Callinan S (2023) Who supports drug-checking services in Australia? An analysis of 2019 National Drug Strategy Household Survey data. Drug and Alcohol Review 42(6): 1553–1558. 37402221 10.1111/dar.13707

[bibr11-13591053251321783] Conner MT Norman P (2015) Predicting and Changing Health Behaviour: Research and Practice With Social Cognition Models. Maidenhead: Open University Press.

[bibr12-13591053251321783] Davis AK Rosenberg H (2016) Using the theory of planned behavior to predict implementation of harm reduction strategies among MDMA/ecstasy users. Psychology of Addictive Behaviors 30(4): 500–508. 27322805 10.1037/adb0000167

[bibr13-13591053251321783] Eassey C Hughes CE Wadds P , et al. (2024) A systematic review of interventions that impact alcohol and other drug-related harms in licensed entertainment settings and outdoor music festivals. Harm Reduction Journal 21(1): 47. 38383344 10.1186/s12954-024-00949-4 PMC10882826

[bibr14-13591053251321783] Fishbein M Triandis HC Kanfer FH , et al. (2001) Factors influencing behavior and behavior change. In: Baum A Revenson TA Singer JE (eds) Handbook of Health Psychology. Mahwah, NJ: Lawrence Erlbaum, pp.3–17.

[bibr15-13591053251321783] Godin G Sheeran P Conner M , et al. (2010) Social structure, social cognition, and physical activity: A test of four models. British Journal of Health Psychology 15(1): 79–95. 19321038 10.1348/135910709X429901

[bibr16-13591053251321783] Hagger MS Hamilton K (2022) Social cognition theories and behavior change in COVID-19: A conceptual review. Behaviour Research and Therapy 154: 104095. 35605335 10.1016/j.brat.2022.104095 PMC9005242

[bibr17-13591053251321783] Hagger MS Hankonen N Kangro EM , et al. (2019) Trait self-control, social cognition constructs, and intentions: Correlational evidence for mediation and moderation effects in diverse health behaviors. Applied Psychology. Health and Well-Being 11(3): 407–437. 30724028 10.1111/aphw.12153

[bibr18-13591053251321783] Hagger MS Luszczynska A (2014) Implementation intention and action planning interventions in health contexts: State of the research and proposals for the way forward. Applied Psychology. Health and Well-Being 6(1): 1–47. 24591064 10.1111/aphw.12017

[bibr19-13591053251321783] Hagger MS Moyers S McAnally K , et al. (2020a) Known knowns and known unknowns on behavior change interventions and mechanisms of action. Health Psychology Review 14(1): 199–212. 31964227 10.1080/17437199.2020.1719184

[bibr20-13591053251321783] Hagger MS Smith SR Keech JJ , et al. (2020b) Predicting social distancing intention and behavior during the COVID-19 pandemic: An integrated social cognition model. Annals of Behavioral Medicine 54(10): 713–727. 32914831 10.1093/abm/kaaa073 PMC7543267

[bibr21-13591053251321783] Hamilton K Smith SR Keech JJ , et al. (2020) Application of the health action process approach to social distancing behavior during COVID-19. Applied Psychology. Health and Well-Being 12(4): 1244–1269. 33006814 10.1111/aphw.12231 PMC7537318

[bibr22-13591053251321783] Hu LT Bentler PM (1999) Cutoff criteria for fit indexes in covariance structure analysis: Conventional criteria versus new alternatives. Structural Equation Modeling: A Multidisciplinary Journal 6(1): 1–55.

[bibr23-13591053251321783] Kaushal N Bérubé B Hagger MS , et al. (2021) Investigating the role of self-control beliefs in predicting exercise behavior: A longitudinal study. British Journal of Health Psychology 24(4): 1155–1175. 10.1111/bjhp.12525 33870633

[bibr24-13591053251321783] Kelly PJ Deane FP McCarthy Z , et al. (2011) Using the theory of planned behaviour and barriers to treatment to predict intention to enter further treatment following residential drug and alcohol detoxification: A pilot study. Addiction Research & Theory 19(3): 276–282.

[bibr25-13591053251321783] Kennedy MC Scheim A Rachlis B , et al. (2018) Willingness to use drug checking within future supervised injection services among people who inject drugs in a mid-sized Canadian city. Drug and Alcohol Dependence 185: 248–252. 29475198 10.1016/j.drugalcdep.2017.12.026

[bibr26-13591053251321783] Kim M Suh D Barone JA , et al. (2022) Health literacy level and comprehension of prescription and nonprescription drug information. International Journal of Environmental Research and Public Health 19(11): 6665. 35682249 10.3390/ijerph19116665 PMC9180079

[bibr27-13591053251321783] Lin CY Imani V Majd NR , et al. (2020) Using an integrated social cognition model to predict COVID-19 preventive behaviours. British Journal of Health Psychology 25(4): 981–1005. 32780891 10.1111/bjhp.12465 PMC7436576

[bibr28-13591053251321783] Maghsoudi N Tanguay J Scarfone K , et al. (2022) Drug checking services for people who use drugs: A systematic review. Addiction 117(3): 532–544. 34729849 10.1111/add.15734 PMC9299873

[bibr29-13591053251321783] McAnally K Hagger MS (2023) Health literacy, social cognition constructs, and health behaviors and outcomes: A meta-analysis. Psychology and Health 42(4): 213–234. 10.1037/hea0001266 37023324

[bibr30-13591053251321783] McEachan RRC Conner M Taylor NJ , et al. (2011) Prospective prediction of health-related behaviors with the theory of planned behavior: A meta-analysis. Health Psychology Review 5(2): 97–144.

[bibr31-13591053251321783] Measham FC (2019) Drug safety testing, disposals and dealing in an English field: Exploring the operational and behavioural outcomes of the UK’s first onsite ‘drug checking’service. International Journal of Drug Policy 67: 102–107. 30541674 10.1016/j.drugpo.2018.11.001

[bibr32-13591053251321783] Milne S Sheeran P Orbell S (2000) Prediction and intervention in health-related behavior: A meta-analytic review of protection motivation theory. Journal of Applied Social Psychology 30(1): 106–143.

[bibr33-13591053251321783] Miron JA (2003) The effect of drug prohibition on drug prices: Evidence from the markets for cocaine and heroin. Review of Economics and Statistics 85(3): 522–530.

[bibr34-13591053251321783] Ng TWY Cowling BJ So HC , et al. (2020) Testing an integrative theory of health behavioural change for predicting seasonal influenza vaccination uptake among healthcare workers. Vaccine 38(3): 690–698. 31668824 10.1016/j.vaccine.2019.10.041

[bibr35-13591053251321783] Olsen A Baillie G Bruno R , et al. (2023) CanTEST health and drug checking service program evaluation. Australian National University Canberra, ACT.

[bibr36-13591053251321783] Piatkowski T Gibbs N Dunn M (2024a) Beyond the law: Exploring the impact of criminalising anabolic–androgenic steroid use on help-seeking and health outcomes in Australia. Journal of Criminology 57(1): 62–82.

[bibr37-13591053251321783] Piatkowski T Dunn M (2024) Navigating Risks and reducing harm: A gendered analysis of anabolic–androgenic steroid users within the risk environment framework. Contemporary Drug Problems 51(2): 111–128.

[bibr38-13591053251321783] Piatkowski T Havnes IA Kill E , et al. (2024b) “The compounds for females are really commonly faked!”: Women’s challenges in anabolic steroid acquisition and the place of drug checking interventions. Drug and Alcohol Review 43(7): 1962–1966. 39187954 10.1111/dar.13931

[bibr39-13591053251321783] Piatkowski T Puljevic C Francis C , et al. (2023) “They sent it away for testing and it was all bunk”: Exploring perspectives on drug checking among steroid consumers in Queensland, Australia. International Journal of Drug Policy 119: 104139. 37481876 10.1016/j.drugpo.2023.104139

[bibr40-13591053251321783] Ritter A (2020) Making drug policy in summer—drug checking in Australia as providing more heat than light. Drug and Alcohol Review 39(1): 12–20. 31774235 10.1111/dar.13018

[bibr41-13591053251321783] Rogers RW (1975) A protection motivation theory of fear appeals and attitude change. Journal of Psychology 91(1): 93–114. 28136248 10.1080/00223980.1975.9915803

[bibr42-13591053251321783] Rolova G Gavurova B Petruzelka B (2021) Health literacy, self-perceived health, and substance use behavior among young people with alcohol and substance use disorders. International Journal of Environmental Research and Public Health 18(8): 4337. 33921885 10.3390/ijerph18084337 PMC8073264

[bibr43-13591053251321783] Rosseel Y (2012) Iavaan: An R package for structural equation modeling. Journal of Statistical Software, 48(2): 1–36.

[bibr44-13591053251321783] Schwarzer R Lippke S Ziegelmann JP (2008) Health action process approach - A research agenda at the Freie Universitat Berlin to examine and promote health behavior change. Zeitschrift Fur Gesundheitspsychologie 16(3): 157–160.

[bibr45-13591053251321783] Sheeran P Klein WM Rothman AJ (2017) Health behavior change: Moving from observation to intervention. Annual Review of Psychology 68(1): 573–600. 10.1146/annurev-psych-010416-044007 27618942

[bibr46-13591053251321783] Sherman SG Morales KB Park JN , et al. (2019) Acceptability of implementing community-based drug checking services for people who use drugs in three United States cities: Baltimore, Boston and Providence. International Journal of Drug Policy 68: 46–53. 30991301 10.1016/j.drugpo.2019.03.003

[bibr47-13591053251321783] Stringhini S Sabia S Shipley M , et al. (2010) Association of socioeconomic position with health behaviors and mortality. Journal of the American Medical Association 303(12): 1159–1166. 20332401 10.1001/jama.2010.297 PMC2918905

[bibr48-13591053251321783] Taylor S Buchanan J Ayres T (2016) Prohibition, privilege and the drug apartheid: The failure of drug policy reform to address the underlying fallacies of drug prohibition. Criminology and Criminal Justice 16(4): 452–469.

[bibr49-13591053251321783] Vederhus J-K Zemore SE Rise J , et al. (2015) Predicting patient post-detoxification engagement in 12-step groups with an extended version of the theory of planned behavior. Addiction Science & Clinical Practice 10(1): 15. 26092327 10.1186/s13722-015-0036-3 PMC4636789

[bibr50-13591053251321783] Wardle F Piatkowski T Clifford S , et al. (2024) Safe beats down under: Investigating the support of drug checking at a regional festival in the Northern Territory, Australia. Drugs Education Prevention and Policy 1–9. 10.1080/09687637.2024.233093840206199

