# Supplementary material for: Psychological and socio-structural determinants of intentions to use drug checking services
Source: J Health Psychol. 2025 Mar 21;30(14):4385–400. doi: 10.1177/13591053251321783 (PMC12678645; doi:10.1177/13591053251321783)
Supplement: sj-docx-1-hpq-10.1177_13591053251321783 – Supplemental material for Psychological and socio-structural determinants of intentions to use drug checking services [file sj-docx-1-hpq-10.1177_13591053251321783.docx]

**Supplementary Documents**

**Appendix A**

*Items and Response Scales for Variables of the Integrated Model*

| Variable | Item(s)/measure | Scale |
| --- | --- | --- |
| Attitude | For me, using a drug checking service would be… | 1 = bad, 7 = good  1 = worthless, 7 = valuable  1 = harmful, 7 = beneficial |
| Subjective norm | Most people who are important to me would approve of me using a drug checking service  Most people whose opinions I value think that I should use a drug checking service  Most people like me would use a drug checking service  Most people like me would think that using a drug checking service is a good thing to do | 1 = strongly disagree, 7 = strongly agree |
| Perceived behavioural control | It is mostly up to me whether I use a drug checking service  I am confident that I can use a drug checking service  I have complete control over whether I use a drug checking service. | 1 = strongly disagree, 7 = strongly agree |
| Risk perceptions | It would be risky for me to use a drug checking service?  If I used a drug checking service, there would be risk involved | 1 = strongly disagree, 7 = strongly agree |
| Health literacy | How confident are you filling out health information forms or medical information forms by yourself?  When you have a medical or health issue, how often do you have problems understanding information you find, or is provided to you by your doctor, about that issue? | 1 = not at all, 5 = extremely 1 = never, 5 = always |
| Drug literacy | Overall Safety (e.g., overdose potential)  Health effects (e.g., headache, vomiting)  Effects on your body (e.g., balance, coordination)  Effects on social skills (e.g., communication, speech, perception) | 1 = not at all, 5 = extremely |
|  |  |  |
| Intention | I intend to use a drug checking service  I expect I will use a drug checking service  It is likely that I will use a drug checking service  I am willing to use a drug checking service | 1 = strongly disagree, 7 = strongly agree |

**Appendix B**

*Participant characteristics (N = 324)*

|  | **Participants (*N*)** | **Percentage (%)** |
| --- | --- | --- |
| ***Age (years)*** |  |  |
| Mean | 22.32 |  |
| SD | 7.21 |  |
| Range | 38 |  |
| ***Gender*** |  |  |
| Male | 104 | 32.32 |
| Female | 214 | 65.85 |
| Non-binary | 6 | 1.83 |
| ***Education Level*** |  |  |
| Junior/Lower/Primary School | 2 | 0.62 |
| Senior/High/Secondary School | 203 | 62.73 |
| Post-School Vocational/Diploma | 58 | 17.90 |
| Undergraduate University Degree | 57 | 17.59 |
| Postgraduate University Degree | 4 | 1.23 |
| ***Employment Status*** |  |  |
| Casual/Part-Time Work | 242 | 74.69 |
| Unemployed/Home Duties | 48 | 14.81 |
| Full-Time Work | 32 | 9.88 |
| Leave without pay | 2 | 0.62 |
| ***Substance Use (Lifetime)*** |  |  |
| Tobacco Products | 85 | 25.93 |
| Alcoholic Beverages | 144 | 44.44 |
| Cannabis | 89 | 27.47 |
| Cocaine | 37 | 11.42 |
| Amphetamine Type Stimulants | 38 | 11.73 |
| Inhalants | 16 | 4.94 |
| Sedatives or Sleeping Pills | 29 | 8.95 |
| Hallucinogens Opioids | 40 | 12.35 |
| Anabolic Steroids | 1 | 0.31 |
| Other substances | 16 | 4.94 |

**Appendix C**

*Correlations and Factor Loadings.*

Correlations among the variables showed meaningful patterns. Health literacy correlated positively with drug literacy (*r* = .250, *p* < .01), demonstrating a significant association between the two constructs. Drug literacy exhibited significant positive correlations with risk perception (*r* = .256, *p* < .01), attitudes (*r* = 0.250, p < 0.01), and norms (*r* = .119, *p* < .05), highlighting the influence of drug literacy on these aspects. Risk perception was inversely correlated with attitudes (*r* = -.566, *p* < .01), norms (*r* = -.424, *p* < .01), and PBC (*r* = -.329, *p* < .01), implying a cautious approach among those perceiving higher risks. Norms were positively correlated with attitudes (*r* = .550, *p* < .01) and PBC (*r* = .424 *p* < .01. Intentions showed a significant positive correlation with attitudes (*r* = 0.506, *p* < .01).

The confirmatory factor analysis model fits supported the adequacy of the measurement model for the examined scales. The comparative fit index (CFI) ranges were 0.882 to 0.991, indicative good model fit with the data. The exploratory factor analysis (EFA) conducted on the drug literacy measures revealed that the first component explains 78.859% of the total variance, with an initial eigenvalue of 3.154. Subsequent components contribute to explaining additional variance, with the second and third components explaining 14.614% and 6.527% respectively. This indicates a significant contribution of drug literacy to the overall understanding of the data, with nearly 79% of the variance explained by this factor alone.
